# The insecticidal effect of the botanical insecticide chlorogenic acid on *Mythimna separata* (Walker) is related to changes in MsCYP450 gene expression

**DOI:** 10.3389/fpls.2022.1015095

**Published:** 2022-10-12

**Authors:** Dong-jiang Lin, Yong Fang, Ling-yun Li, Li-zhao Zhang, San-ji Gao, Ran Wang, Jin-da Wang

**Affiliations:** ^1^ National Engineering Research Center for Sugarcane, Fujian Agricultural and Forestry University, Fuzhou, China; ^2^ Hunan Agricultural Biotechnology Research Institute, Hunan Academy of Agriculture Science, Changsha, China; ^3^ Institute of Plant Protection, Beijing Academy of Agriculture and Forestry Sciences, Beijing, China

**Keywords:** chlorogenic acid, sublethal effect, P450, RNAi, *mythinma seperata*

## Abstract

The oriental armyworm *Mythimna separata* (Walker) (Lepidoptera: Noctuidae) can feed on the leaves of many crops, resulting in vast areas of damage and severe losses. Therefore, this insect has become a significant agricultural pest in north Asia. In this study, we fed 3^rd^ instar larvae with artificial diets containing different concentrations of chlorogenic acid and found a significant lethal effect and the mortality increased with increasing chlorogenic acid concentration. Next, we measured the sublethal effect of chlorogenic acid at LC_20_ on the growth and development of *M. separata* larvae. The durations of the 4^th^ and 5^th^ instar were longer than those of the control group (prolonged by 0.8 and 0.6 days, respectively), and the 6^th^ instar was shorter (by 1.1 days). The total survival rate, pupation rate, eclosion rate, sex ratio, and oviposition amount in the LC_20_ chlorogenic acid-treated group were significantly lower than those in the control group. Furthermore, transcriptome analysis of 3^rd^ instar larvae fed various concentrations of chlorogenic acid revealed that several *MsCYP450* genes were significantly up-regulated, and this finding was further validated by qRT-PCR. In addition, various concentrations of chlorogenic acid and different treatment times significantly affected the enzyme activity of CYP450 in 3^rd^ instar larvae. Importantly, dietary ingestion of dsMsCYP450 significantly reduced the mRNA level of *MsCYP450* genes and increased mortality in the presence of chlorogenic acid. Our results revealed that *MsCYP6B6*, *MsCYP321A7*, and *MsCYP6B7-like* play an essential role in the detoxification of chlorogenic acid by *M. separata*. This study provides evidence of control effect by botanical insecticide chlorogenic acid on *M. separata*, and potential detoxification mechanism mediated by P450 of botanical insecticide in arthropods.

## 1. Introduction

Through long-term co-evolution, insects and plants have formed a relatively stable ecological relationship. Plants not only provide nutrients for phytophagous insects, but also initiate a series of physical and chemical defenses to resist insect feeding. Generally, these defense mechanisms can be divided into two categories: constitutive defense and induced defense (Fornoff and Gross, 2014; Chen, 2015; Fyllas et al., 2022; Sobhy et al., 2022). Both defense mechanisms mainly include physical and chemical defenses. Physical defenses specifically include plant morphology, leaf thickness, and fluff, which negatively impact insect feeding behavior (Gary et al., 2006; Whitehill et al., 2016). Chemical defense refers to physiological and biochemical changes, such as induction of plant phytohormone signaling, a decrease in nutritional components, and production of defense proteins and plant secondary metabolites (Ahn et al., 2007; Body et al., 2019; Shen et al., 2021; Divekar et al., 2022). However, insects have also evolved several behaviors to overcome plant defenses, such as changing feeding strategies and regulating growth rhythm and development. In addition to these behavioral changes, biochemical and molecular characteristics also contribute greatly to adaptation to plant defense systems. For example, phytophagous insects regulate the composition, quantity, and quality of digestive enzymes to overcome the protease inhibitors in plants (Cloutier et al., 2000). Other strategies, such as the inhibition of plant defense injury signals and the detoxification to plant secondary substances, also enable insects to escape from the plant defense system.

Insect cytochrome P450 (CYP450) is a terminal oxidase in the multifunctional oxidase system, and it has a catalytic activity on various substrates. Metabolic resistance is an important mechanism underlying insect resistance to traditional insecticides, and CYP450 plays a crucial role in the detoxification of endogenous and exogenous toxic compounds because of its broad-spectrum substrate specificity (Schuler, 2012; Bao et al., 2016; Xu et al., 2020). It has been reported that plant secondary metabolites and insecticides can induce insect CYP450 gene expression; for example, coumarin can induce overexpression of CYP6B2, CYP6B6, and CYP6B7 in *Helicoverpa armigera* and reduce the sensitivity of the insect to methomyl (Chen et al., 2018). Gossypol can induce high expression of CYP6AB14 and CYP9A98 in *Spodoptera exigua*, and after RNA interference (RNAi)-mediated silencing of these genes, larvae are more sensitive to deltamethrin (Hafeez et al., 2019).

The oriental armyworm, *Mythimna separata* (Walker), is a serious polyphagous and migratory insect pest with strong adaptability, and it shows a preference for high-temperature and -humidity environments (Jiang et al., 2011; Kong et al., 2019). Local outbreaks have caused significant damage to crops, such as corn, rice, and sugarcane (Mishra et al., 2021; Yang, 2021). In addition, some economically important crops such as cotton, beans, and vegetables have also suffered damage (Krempl et al., 2021). The long-term continuous cropping of gramineous crops will increase the probability of crop pests and diseases of *M. separata* (Pang et al., 2021). Chemical treatments are the most common and effective method to control *M. separata* (Wang et al., 2018). However, frequent use of pesticides often leads to serious environmental problems and insecticide resistance (Song et al., 2017). Therefore, there is an urgent need to develop new methods and materials with low toxicity to beneficial organisms and high specificity for target insects.

Plant secondary metabolites, including phenolics and flavonoids, play essential roles in insect resistance (Chen et al., 2015; Chen et al., 2018; Xu et al., 2019). The results from various studies have demonstrated that plant phenolic metabolites such as chlorogenic acid (CGA), methyl jasmonate, and tannic acid negatively affect insect feeding behavior, growth, development, and reproduction, and they may have lethal effects on specific insects (Kundu and Vadassery, 2019; Li et al., 2019; Lin et al., 2021). CGA (C16H18O9) is a dihydroxy phenolic compound that is a common secondary metabolite in plants, including higher dicotyledons and ferns (Xi et al., 2014). CGA has been shown to be involved in plant chemical defenses against insect herbivores (Kundu and Vadassery, 2019); for example, it can be used as a resistance factor for thrips in chrysanthemum (Leiss et al., 2009), and induced biosynthesis of CGA in sweet potato confers resistance against sweet potato weevil (Liao et al., 2020). In addition, CGA is the main component in the anti-insect defenses of *Vernonia anthelmintica* Willd (Liu et al., 2020). Besides protecting plants from herbivores, CGA is also involved in plant growth and development processes, such as shoot organogenesis and fruit ripening (A. Neşe Çokuğraş, and Ebru, 2003; Liu, 2016). In our previous research, we found that the attack of M. separata on sugarcane induced significant accumulation of CGA and that CGA has lethal effect on larvae (Wang et al., 2021). Therefore, CGA is a promising environmentally friendly insecticide that is safer for biological use compared with traditional synthetic pesticides.

Although several studies have focused on CGA-mediated plant chemical defenses against insects and the lethal effect of CGA on target insects (Kundu and Vadassery, 2019; Pan et al., 2020), there is no solid evidence of the role of CGA in inhibiting herbivore attack, and the sublethal effects of CGA on insect development and reproduction remain to be determined. In addition, the effect of the CGA regulatory mechanism on an insect is poorly understood. This study aimed to elucidate the lethal and sublethal effects of CGA on *M. separata* larval growth and development. The effect of CGA on detoxification enzyme activity in *M. separata* and the potential key detoxifying genes were investigated by RNA sequencing (RNA-seq). The findings of this study provide the basis for further understanding the detoxification mechanism of CGA in arthropods and a new method in the management of pests with P450-mediated resistance.

## 2. Materials and methods

### 2.1 Insects

*M. separata* larvae were raised in the lab of National Engineering Research Center for Sugarcane, Fujian A&F University, in a controlled temperature (26 ± 1°C) and fixed photoperiod (L16:D8). Preparation of artificial feed and feeding were performed using the feeding method of Lepidoptera insects described by Cao et al. (Cao et al., 2014).

### 2.2 Bioassays

In this study, the concentrations of CGA (purchased from Beijing Solarbio Science & Technology Co., Ltd., purity ≥98%) in the artificial diet were 5mg/mL, 10mg/mL, 20mg/mL, 40mg/mL, and 80mg/mL. During the preparation of the artificial diet, all the main materials were mixed under liquid conditions. To prepare the 80mg/mL CGA artificial diet, 0.4 g of CGA was dissolved in 5 mL of 25% absolute ethanol at room temperature, and 15 g of artificial diet was added. The other artificial diets with different concentrations of CGA were prepared in the same way with the appropriate amounts of CGA, and the artificial diet supplemented with 5 mL of 25% absolute ethanol was used as the control. A piece of the artificial diet was placed in each well of a 24-well plate, and one pre-starvation (12 h) 2^nd^ instar larva was placed on the surface of the diet. Three replicates of 24 larvae were tested for every concentration. The same treatment was also for 3^rd^ and 4^th^ instar larvae. Feeding conditions were the same as those in section 2.1. Each day the artificial diet was checked for freshness, and stale food was replaced, and the death of larvae was recorded. The experiment was terminated after five days of treatment, and the statistical data were collected. Larvae were considered dead when they did not respond when stimulated with an ink brush. The LC_50_ for each treatment was determined by Probit analysis in SPSS 18.0.

### 2.3 Sublethal effects of CGA on the larval development, eclosion rate and fecundity of *M. separata*

The 3^rd^ instar larvae were collected to determine the sublethal effects of CGA on larval growth and development parameters as described by Wang et al. (Wang et al., 2014). LC_20_ was chosen as the concentration for CGA treatment because it resulted in a specific amount of mortality. Healthy 3^rd^ instar larvae were starved for 12 h, then a single 3^rd^ instar larva was placed on the surface of the artificial diet containing the LC_20_ dose of CGA in an individual 25 ml plastic cup and sealed with a lid. Each treatment was performed with 30 larvae, and the treatment was replicated three times. Similarly, twenty 3^rd^ instar larvae were placed on the artificial diet without CGA as a control group, and the treatment was repeated three times. Larvae were examined every day till pupation, and the developmental stage, including molting, pupation, and death, of the larvae was recorded every day. Then we determined the sex of each *M. separata* by pupa and calculated the sex ratio (Chen et al., 2019). After eclosion, the male and female adults were paired and transferred to a cage for mating. After eggs were laid, the egg masses were counted. The date was analysis by t-test in SPSS 18.0.

### 2.4 Transcriptome analysis

The artificial diets with CGA at LC_20_, LC_50_, and LC_80_ were fed to healthy 3^rd^ instar larvae for 5 days, then four to six surviving larvae were randomly selected for further transcriptome analysis with three biological replicates. RNA isolation, cDNA synthesis, library construction, and Illumina sequencing were all performed at Berry Genomics Co., Ltd. (Beijing, China) (Hansen et al., 2010). The RNeasy Micro Kit (Qiagen, Hilden, Germany) was used to isolate total RNA from each sample. RNA purity and concentration were then examined using the NanoDrop 2000, and RNA integrity and quantity were measured using the Agilent 2100 system. Next, an NEB library was established for each sample using mRNA as a template. All libraries were pooled together and subjected to Illumina sequencing with paired-end sequencing. Trinity was used to assemble clean reads, Benchmarking Universal Single-Copy Orthologs (BUSCO) was used to evaluate the integrity of transcript assembly, and Corset program transcripts were used for hierarchical clustering. All unigenes were obtained after assembly, and unigene functional annotation was based on the non-redundant protein sequence (Nr), nucleotide sequence (Nt), protein families (PFAM), KOG and Swiss-Prot databases. Open reading frames (ORFs) were predicted by TransDecoder software with the default setting. Then, paired-end reads were aligned to the unigene sequence using bowtie, and RSEM was used to count the number of reads mapped to each gene and estimate gene expression levels. Differential expression was analyzed using EdgeR. P-values of the results were adjusted to control for the false discovery rate. Genes with |log2 (Fold Change)| > 1 and q value < 0.05 were designated as differentially expressed. Finally, Gene ontology (GO) and Kyoto Encyclopedia of Genes and Genomes (KEGG) enrichment analyses of differentially expressed gene (DEG) sets were performed using GOseq R and KOBAS 3.0, respectively. GO terms with an adjusted p-value below 0.05 were considered significantly enriched in DEGs.

### 2.5 Identification and bioinformatics analysis of CYP450 genes

The assembled unigenes were used as queries in searches against the Nr database with a cut-off E-value < 1.0 E^−5^. The unigenes found in the same BLAST search or that shared high homology with other unigenes were regarded as allelic variants or as different parts of the same gene. The gene with hit result of CYP450 was screened from the Nr results after BLAST. All CYP450 genes of *M. separata* were identified by sequence alignment, and the amino acid sequences were aligned using the default settings in ClustalW 2.0. Then, the CYP450s genes of *M. separata* were compared with those of *H. armigera* and *Spodoptera litura* (obtained from InsectBase 2.0) by performing phylogenetic analysis in MEGA-X.

### 2.6 Enzyme assays of P450 monoxygenases in *M. separata* larvae treated with CGA

Healthy 3^rd^ instar larvae were starved for 12 h, then transferred to an artificial diet with different concentrations (LC_20_, LC_50_, and LC_80_) of CGA. Feeding conditions were the same as those in section 2.1. Healthy 3^rd^ instar larvae were fed an artificial diet with 25% absolute ethanol as a control. After feeding for 1, 3, 5, and 7 days, ten surviving larvae were randomly selected with three replicates per group to analyze the effects of different concentrations of CGA in the artificial diet and different treatment times on P450 enzyme activities using the CYP450 enzyme assay kit (CK-E93532, Shanghai Enzymatic Biotechnology Co., Ltd.) according to the kit instructions.

### 2.7 Validation of expression profiles using qRT-PCR

RNA-seq analysis and assays of detoxification-related CYP450 protein activities in *M. separata* treated for different times with different concentrations of CGA revealed that *MsCYP450* genes and CYP450 detoxification proteins were significantly up-regulated. Therefore, we selected seven *MsCYP450* genes that were up-regulated considerably in response to different concentrations of CGA for validation by qRT-PCR using the same RNA that was used in RNA-seq. Primer 5 was used to design specific primer pairs (**Table S1**), and primers were synthesized by Tsingke Biotechnology Co., Ltd. in China. The cDNA synthesis reaction was performed using the HiScript^®^ II Q RT SuperMix kit with gDNA wiper (Vazyme, China) according to the manufacturer’s protocol using 1 μg of total RNA as a template per reaction. QRT-PCR was performed with Hieff^®^ qPCR Green Master Mix (Yeasen, China). Finally, data were analyzed using the 2^-ΔΔCT^ method (Sun et al., 2017), and EF-1α was used as a control to correct for sample-to-sample variation. Three technical replicates were performed for each replicate, and the data were expressed as mean ± standard error (SE).

### 2.8 RNAi in *M. separata*

Fragments of *MsCYP321A7*, *MsCYP6k1-like*, *MsCYP6B6*, *MsCYP324A1*, *MsCYP4V2-like*, *MsCYP6B7-like*, *MsCYP6AE88*, and green fluorescent protein gene (*GFP*) were amplified by PCR using specific primers (**Table S2**) conjugated with the T7 RNA polymerase promoter (TAATACGACTCACTATAGGG). The T7 Ribomax ™ Express RNAi System (Promega, Madison, WI, USA) was used to synthesize double-stranded RNAs (dsRNAs) as described in the manual. Thirty *M. separata* larvae at the late 2^nd^ instar stage were fed an artificial diet containing 100 μg dsRNA in a 24-well plate for 3 days, and fresh dsRNA was added every day. The control group was treated with the same amount of ddH_2_O. After 3 days, five living larvae in each group (control, *MsCYP321A7-dsRNA*, *MsCYP6k1-like-dsRNA*, *MsCYP6B6-dsRNA*, *MsCYP324A1-dsRNA*, *MsCYP4V2-like-dsRNA*, *MsCYP6B7-like-dsRNA*, *MsCYP6AE88-dsRNA*, and *GFP-dsRNA*) were collected for total RNA extraction for determination of gene expression. The approximately 25 larvae remaining in each treatment were used for bioassays with CGA at LC_50_ as described above (section 2.3). The number of dead larvae was recorded after CGA application for 6 days. The experiment was replicated three times. After dsRNA treatment with the same method, the growth and development period were determined by 2.3 method that using LC_20_ CGA mixed artificial diet.

## 3. Results

### 3.1 The effects of CGA on *M. separata*

The toxicity of CGA against *M. separata* larvae was determined using the feeding method. The mortality was calculated after feeding the 2^st^, 3^rd^ and 4^th^ instar larvae with an artificial diet containing various concentrations of CGA for 5 days. The results (**Table S3**) showed that the mortalities of 3^rd^ larvae fed diets with different concentrations of CGA were significantly different after 5 days (P<0.05). The higher the concentration of CGA, the higher the mortality. The mortality rate of the 5mg/mL CGA treatment group was 13.33% ± 6.67%, while the mortality rate of the 80mg/mL CGA treatment group reached 80.00% ± 13.33%. Based on the bioassay results, 74.48 mg/mL CGA (LC_80_ dose), 26.29 mg/mL CGA (LC_50_ dose) and 7.15 mg/mL CGA (LC_20_ dose) for 3^rd^ larvae were used for further treatment of larvae.

### 3.2 Effects of CGA on the growth and development of larvae

The LC_20_ concentration 7.15 mg/mL was used to assess the sublethal effects of CGA on *M. separata* development and reproduction. The duration of development for each instar is shown in **Table 1**. The results showed that there was no significant difference in the duration of the 3^rd^ instar stage of larvae treated with LC_20_, but there were significant differences in the durations of the 4^th^ and 5^th^ instars, which were prolonged by 36.87% (0.8 days) and 38.22% (0.6 days), respectively (P < 0.0001). However, the duration of the 6^th^ instar larval stage was significantly shortened by 18.15% (1.1 days), and the overall developmental duration of the 3^rd^ to 6^th^ instars was extended by about 0.5 days. Measurement of other growth and development indices of larvae showed that the total survival rate of larvae (90%), eclosion rate (34.09%), sex ratio (0.86), and the number of eggs laid per female (427.8 ± 48.88) of the CGA treatment group were significantly lower than those of the control group. It can be seen that CGA harms the growth, development, and reproduction of larvae.

**Table 1:** Sublethal effects of CGA at LC_20_ on growth and developmental indices of *M. separata*.

Index		Treatments	
		CK	Chlorogenic acid
Developmental duration (days)	3^rd^ instar larva	3.52 ± 0.01^ns^	3.56 ± 0.03
	4^th^ instar larva	2.17 ± 0.03	2.97 ± 0.07****
	5^th^ instar larva	1.57 ± 0.02	2.17 ± 0.06****
	6^th^ instar larva	7.03 ± 0.09****	5.95 ± 0.09
Larval duration from the 3^rd^ instar (days)		14.27 ± 0.10	14.75 ± 0.11**
Total survival rate of larvae (%)		98%**	90%
Pupation rate (%)		100%^ns^	97.78%
Eclosion rate (%)		44.9%**	34.09%
Sex ratio (♀/♂)		2.14****	0.86
Number of eggs laid per female		524.8 ± 80.02****	427.8 ± 48.88

Data in the table are mean ± SE. Significance level(t-test):**p<0.01, ****p<0.0001, ns: not significant.

### 3.3 Transcriptome analysis of *M. separata*

To assess the potential mechanism underlying the lethal effects of CGA on *M. separata* and potential detoxification pathways, RNA-seq was carried out to identify genes encoding target proteins and potential insecticide detoxification enzymes.

Twelve *M. separata* libraries were sequenced on the Illumina platform and pooled together for assembly. All reads were cleaned, and Trinity was used to conduct quality checks. A total of 310,336,443 reads were assembled into 257,014 transcripts with an N50 length of 1,892. The contigs were assembled into 134,240 unigenes with an average length of 1,176 bp (**Table 2**) using paired-end joining and gap-filling methods. The length distribution was mainly between 300 and 500 bp (35.66% of sequences); there were no sequences < 300 bp, and 14.91% of sequences were longer than 2 kb (**Figure S1**).

**Table 2:** An overview of the Illumina sequencing of the *M. separata* transcriptome.

Parameter	Value
Total number of raw reads	322,402,739
Total number of clean reads	310,336,443
Total number of clean bases	85.47G
GC percentage	47.78%
Number of transcripts	257,014
Total transcript nucleotides	302,148,057
Mean length of transcripts (bp)	1,176
Number of unigenes	134,240
Total unigene nucleotides	128,131,440
Mean length of unigenes	954
Annotated in NR	27,039
Annotated in NT	30,108
Annotated in KEGG	14,852
Annotated in SwissProt	13,607
Annotated in PFAM	21,624
Annotated in GO	21,623
Annotated in KOG	7,329

To annotate unigenes, a BLASTX search of the Nr protein database of the National Center for Biotechnology Information (NCBI), was performed with a cut-off E-value of 10^−5^. A BLAST hit was obtained for 27,039 distinct sequences (20.1% of the total). Sequences were also used as queries in searches against several other databases, including the Nt, Swiss-Prot, PFAM protein, GO, and KOG databases (**Table 2**). Based on the best hit in the Nr database, 7300 (27.0%) annotated unigenes had the highest homology to sequences in *H. armigera*. In comparison, fewer matched sequences in *S. litura* (19.7%) and *Heliothis virescens* (16.5%). The fewest sequences matched hits in the more distantly related species *Trichoplusia ni* (6.3%) and *Chilo suppressalis* (5.5%) (**Figure S2**).

**Figure 1:**
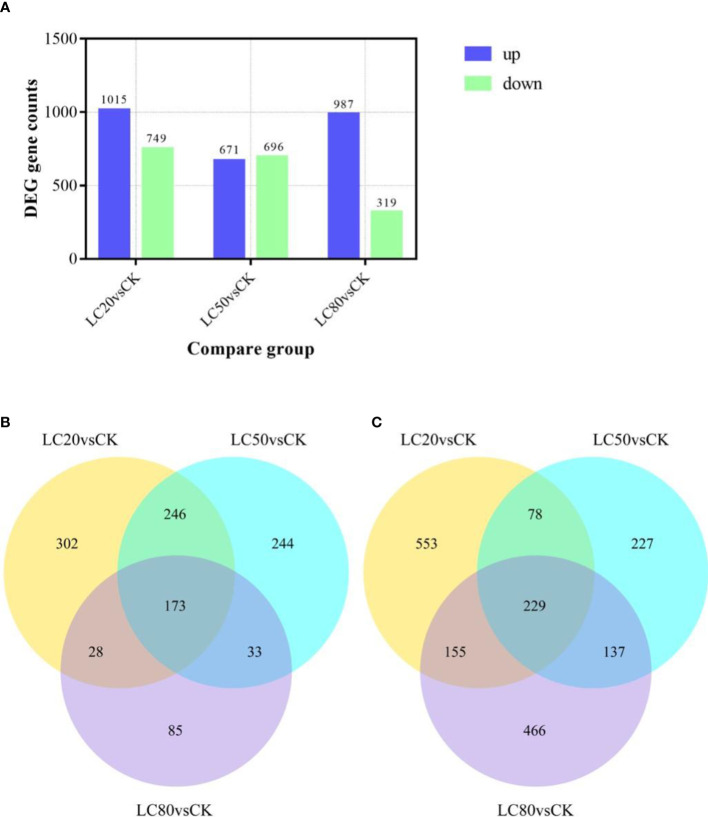
The number of DEGs in *M. separata* larvae treated with different concentrations of CGA. **(A)** The number of DEGs in different treatment. The number in the column indicates the number of DEGs. **(B)** The number of DEGs up-regulated by all treatments. **(C)** The number of DEGs down-regulated by all treatments.).

Expression levels of genes in *M. separata* treated with artificial diets containing one of three different concentrations of CGA (LC_20_, LC_50_, and LC_80_) were compared with those of genes in the control group (CK). The comparison LC_20_vsCK had the most significant DEGs, 1764 (1015 up-regulated and 749 down-regulated). LC_80_vsCK had the least number of DEGs, of which 967 were up-regulated and 319 were down-regulated. There were 671 up-regulated DEGs and 696 down-regulated DEGs identified in the LC_50_vsCK comparison (**Figure 1A**). Analysis of the intersection of DEGs revealed that 229 genes were up-regulated and 173 genes were down-regulated in response to all three CGA treatments (**Figures 1B, C**).

**Figure 2:**
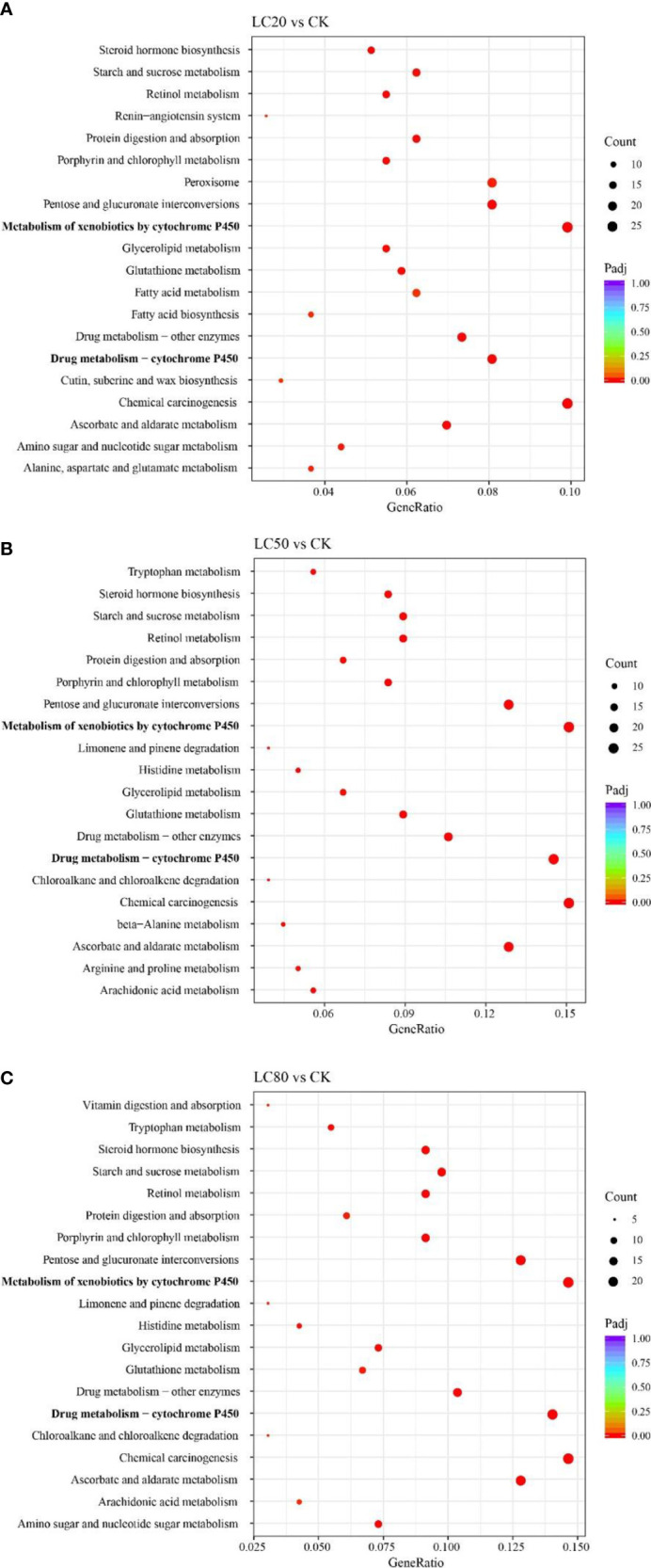
KEGG enrichment analyses of DEGs from different comparisons. **(A)** LC_20_vsCK comparison. **(B)** LC_50_vsCK comparison. **(C)** LC_80_vsCK comparison.

GO and KEGG enrichment analyses were performed for all DEGs to understand the possible mechanisms underlying gene expression differences between control and CGA-treated *M. separata* larvae. DEGs from the LC_20_vsCK, LC_50_vsCK, and LC_80_vsCK comparisons were all mainly enriched in the GO biological process (BP) term transmembrane transport, the cellular component (CC) term extracellular region, and the molecular function (MF) term oxidoreductase activity (**Figure S3**). In KEGG enrichment analysis, DEGs from LC_20_vsCK, LC_50_vsCK, and LC_80_vsCK were all enriched primarily in the metabolism of xenobiotics by cytochrome P450, drug metabolism - cytochrome P450, and chemical carcinogenesis (**Figure 2**).

### 3.4 Analysis of the P450 genes responding to CGA in *M. separata*

**Figure 3:**
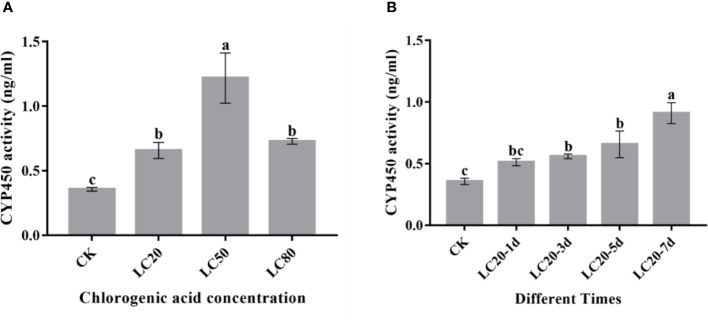
The activity of CYP450 proteins in the 3^rd^ instar larvae of *M. separata* fed artificial diets containing CGA. **(A)** Artificial diet containing different concentrations of CGA; **(B)** Artificial diet containing CGA at LC_20_ with different treatment times. Different letters above the bars show significant differences between groups according to Tukey’s multiple comparisons tests at P < 0.05. Error bars represent the standard deviation (SD) of the means.).

P450 enzyme activity assays showed that P450 activity was induced by different concentrations of CGA (**Figure 3A**). P450 activity significantly increased with increasing CGA concentration and reached the maximum at LC_50_. In addition, we also assessed the effect of duration of LC_20_ CGA treatment on *M. separata* P450 enzyme activity (**Figure 3B**). The P450 enzyme activity increased significantly post-treatment and reached the maximum at 7 days.

**Figure 4:**
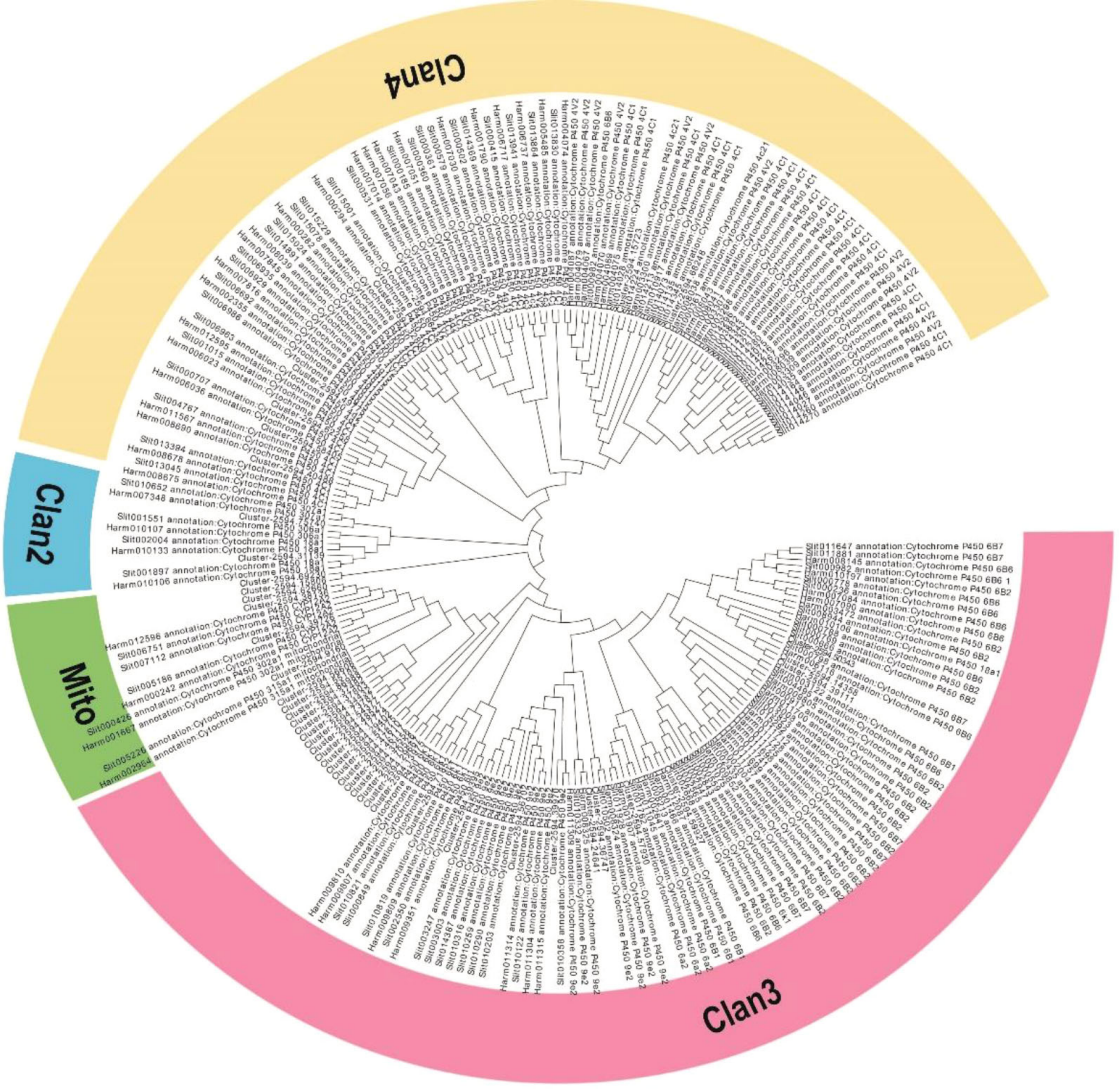
Phylogenetic analysis of the CYP450 genes from *M. separata*, *H. armigera*, and *S. litura*. The tree was constructed from multiple sequence alignments using MEGA-X software.

From our transcriptome data, 139 sequences encoding CYP450s were identified, and these sequences corresponded to 61 non-redundant unigenes. Of these, 44 CYP450 genes encoding proteins with more than 200 amino acids identical to annotated CYP450 proteins were used for further analysis (**Table S4**). The lengths of these CYP450 genes ranged from 662 to 4858 bp. Then, from phylogenetic tree analysis, the CYP450 genes were categorized into four CYP450 (CYP) clans: CYP2, CYP3, CYP4, and the mitochondrial clan (**Figure 4**). Twenty-eight genes were assigned to the CYP3 clan, which was the most prominent clan; the CYP4 clan was the second largest with 8 genes; 2 genes were assigned to the CYP2 clan; and 6 genes were assigned to the mitochondrial clan, which is only found in animals (**Figure 4**).

**Figure 5:**
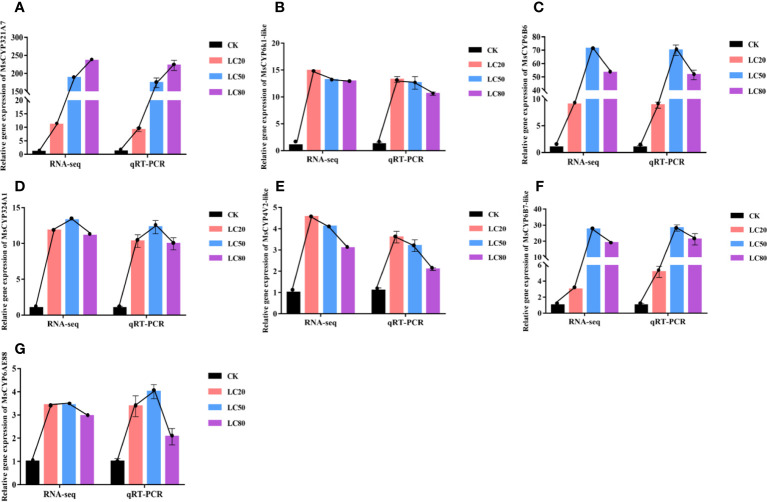
Comparison of relative fold change determined from RNA-seq and qRT-PCR analysis of the same samples. (From **A**–**G** the genes are *MsCYP321A7*, *MsCYP6k1-like*, *MsCYP6B6*, *MsCYP324A1*, *MsCYP4V2-like*, *MsCYP6B7-like*, and *MsCYP6AE88*, respectively).

To verify the accuracy of the expression profiles obtained by RNA-seq, we used the same RNA sample as template in qRT-PCR analysis to determine the expression levels of seven *MsCYP450* DEGs. As shown in **Figure 5**, the RNA-seq expression patterns of the DEGs were similar to those determined by qRT-PCR. After treatment with CGA at LC_20_, LC_50_ and LC_80_, gene expression was up-regulated. Of the *MsCYP450* genes, *MsCYP321A7* was the most significantly up-regulated. *MsCYP6k1-like*, *MsCYP324A1*, and *MsCYP6AE88* were all expressed at high levels after treatment with different concentrations of CGA. *MsCYP4V2-like* was the most highly expressed after treatment with CGA at LC_20_, and its expression then decreased with increasing CGA concentration. We also found that the seven *MsCYP450* genes had high expression levels after being treated with CGA at LC_50_. This is consistent with the observation of the highest activity of CYP450 detoxification enzymes in *M. separata* treated with CGA at LC_50_.

**Figure 6:**
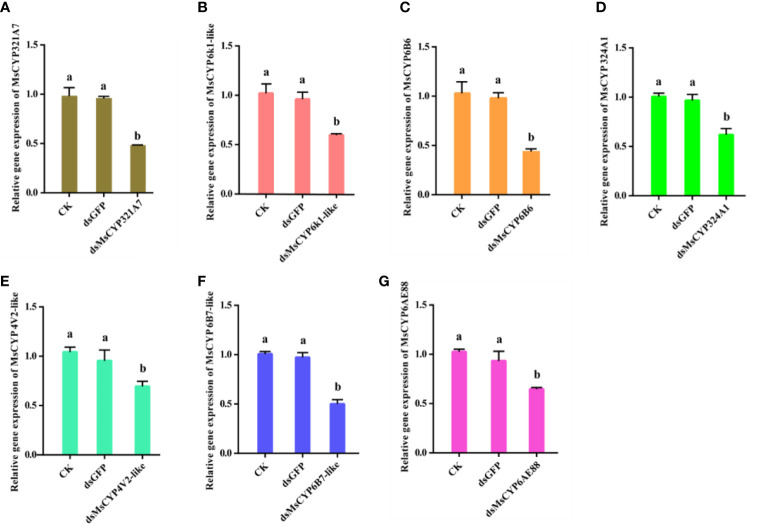
The relative gene expression levels of *MsCYP450* genes after dsMsCYP450 gene treatment for 3 days. (From **A**–**G**, the genes are *MsCYP321A7*, *MsCYP6k1-like*, *MsCYP6B6*, *MsCYP324A1*, *MsCYP4V2-like*, *MsCYP6B7-like*, and *MsCYP6AE88*, respectively. Different lowercase letters above the bars indicate a significant difference (p < 0.05) based on one-way ANOVA followed by Tukey’s HSD test for multiple comparisons. Means ± SE from three replicates are shown).

**Figure 7:**
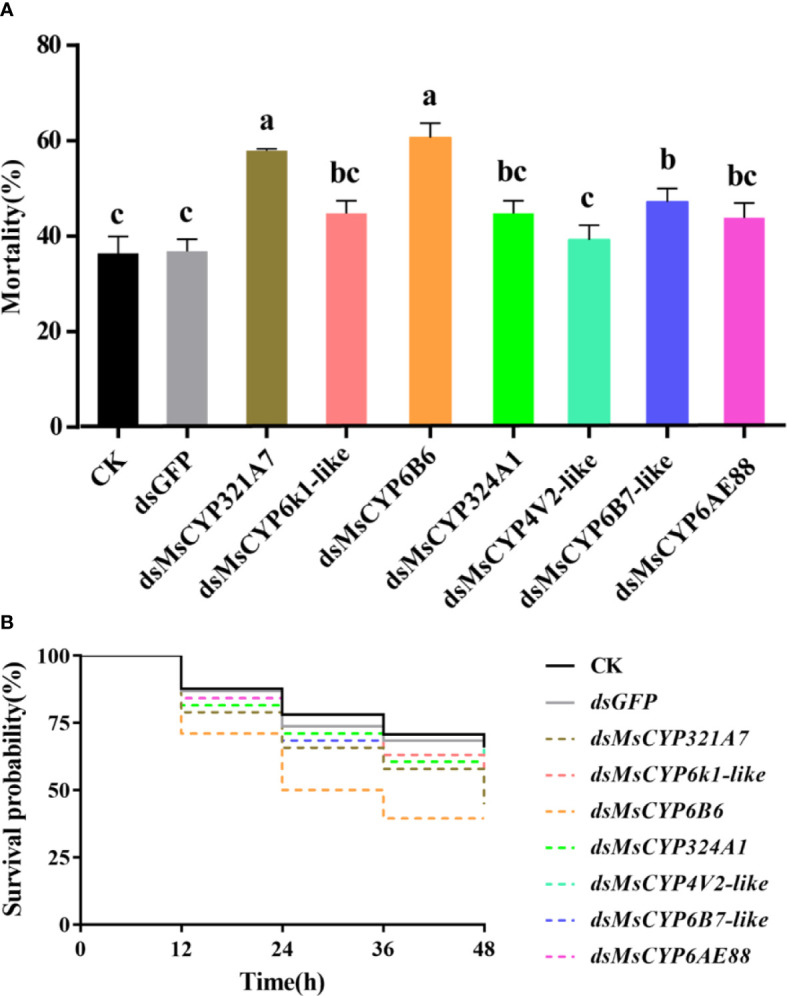
The larval mortality and survival rates for *M. separata* larvae after dsRNA treatment. **(A)** Larvae were continuously fed dsRNA for 3 days, and mortality was evaluated for 2 days; **(B)** The survival rates of larvae scored for 2 days after dsMsCYP450 gene treatment for 3 days. Different lowercase letters (a, b and c) above the bars indicate significant differences (p < 0.05) based on one-way ANOVA followed by Tukey’s HSD test for multiple comparisons. Means ± SE from three replicates are shown).

After continuous ingestion of dsGFP and dsMsCYP450 genes for three days, the late 2^nd^ instar molted to the 3^rd^ instar. Few larvae died after being fed dsRNA, but the level of *MsCYP450* gene expression decreased significantly (43.89%–69.39%) compared with the control group (**Figure 6**). The lowest expression level was found in larvae fed dsMsCYP6B6, with a 56.11% reduction in expression. Next, approximately 25 surviving larvae exposed to CK (ddH_2_O), dsGFP, or dsMsCYP450 genes were used for further bioassay experiments. After 2 days, larval mortality among the dsMsCYP6B6, dsMsCYP321A7, and dsMsCYP6B7-like treatment groups (60.71%, 57.94%, and 47.22%, respectively) was much higher than that in the CK and dsGFP treatment groups (36.45% and 36.90%, respectively, **Figure 7**). The larval mortality in the dsMsCYP6k1-like, dsMsCYP324A1, dsMsCYP4V2-like, and dsMsCYP6AE88 groups was not significantly different compared with that in the CK and dsGFP treatment groups. After 5 days, the largest larval mortality was dsMsCYP6B6 treatment groups reached 78.97% (**Figure S4**).

In addition, we also tested larvae growth and development parameter of *M. separata* larvae after different dsRNA treatment showed that (**Table S5**). Compared with the control group, the 3^rd^ instar duration of the treatment groups was not significantly different, while the 4^th^ and 5^th^ instars began to be significantly prolonged. The longest of 4^th^ instar duration was dsMsCYP321A-7 treatment (3.19 days) and the 5^th^ instar duration was dsMsCYP6B7-like treatment (2.53 days). Compared with the control group, the 6^th^ instar duration of the treatment groups were all shorten and the shortest was dsMsCYP6B6 treatment (5.32 days).

## 4. Discussion

### 4.1 Analysis of the insecticidal activity of CGA against *M. separata* larvae

In this study, the effects of different concentrations of CGA on *M. separata* larvae were investigated by adding different concentrations of CGA to an artificial diet. The larvae in the CGA treatment group started to die after 3 days, and peak death was observed between days 3 and 5. This indicates that CGA has no acute insecticidal effect on *M. separata* larvae within the experimental concentration range of this study, but with prolonged feeding time, CGA may accumulate in larvae and have an insecticidal effect. Similar results have been obtained when treating 3^rd^ instar larvae of *Plutella americana* with 0.500% CGA. The mortality of 5^th^ instar larvae of *P. americana* in the treatment group increased with increasing CGA concentration in the artificial diet and was significantly higher than that in the control group (Pan et al., 2020). Similar results were also obtained when 2^nd^ instar larvae of *Plutella viridis* were treated with 0.3% CGA. The mortality rate increased rapidly from 20% to 50% after 20 days of treatment and reached 100% after 34 days of treatment (Wang et al., 2014).

Generally speaking, botanical insecticides are thought to be eco-friendly and relatively safe. These insecticides have the following main properties: pest selectivity, low risk to non-target organisms, biodegradability, and low risk of inducing insect resistance (Benelli et al., 2019; Zainab and Manfred, 2020). In recent years, many studies have concentrated on the use of plant extracts, particularly biologically active compounds of plant-derived and essential oils, as potential alternatives to commercial insecticides (Isman and Grieneisen, 2014). Therefore, it is necessary to pay attention to the excavation and use of botanical pesticides. Other phenolic substances have shown insectidal activity; for example, 50 μg/mL kaempferol treatment for 72 h, caused 82% mortality of 4^th^ instar larvae of *Culex quinquefasciatus* (Huang et al., 2014); 10 mg/mL periplocosides treatment for 24 h resulted in 76.2% mortality of *Schizaphis graminum* (Rondani) and 37.5% mortality of *M. separata* (Li et al., 2019). The essential oils of *Schinus areira* and *Thymus hyemalis* (in the family Lamiaceae) had insecticidal activity against *Rhipibruchus picturatus* (F.) (Coleoptera: Bruchinae) and *Eceratoniae ceratoniae* Zeller (Lepidoptera: Pyralidae), respectively (Mattar et al., 2022; Adouane et al., 2022).

### 4.2 Analysis of the sublethal effect of CGA on *M. separata* larvae

Studies to date have shown that CGA has adverse effects on the growth and development of insects and can even be oxidized to more toxic quinones in insects, which have a direct poisoning effect. (Hu et al., 2009; Kundu and Vadassery, 2019) In this study, feeding the 3^rd^ instar larvae with a LC_20_ CGA diet prolonged the duration of *M. separata* larval development (instars 4–5), probably due to a fitness penalty from resisting CGA. Part of the energy intake is used for growth and development, while the other part is used for detoxification metabolism of CGA. But the developmental duration of the mature larvae (6^th^ instar) was shortened, which may be related to the tendency of Lepidoptera to survive poor environments as pupae and the premature pupation of the mature larvae caused by CGA treatment (Deng, 2018). This result was similar to that of previous studies in Lepidoptera, which showed that the duration of *Helicoverpa zea* development was significantly prolonged after ingesting artificial diets containing CGA and caffeic acid (Summers and Felton, 1994), and that CGA reduces the growth, development, and fecundity of *Hyphantria cunea* larvae (Pan et al., 2020). All the results mentioned above show that CGA can prolong the growth period of insects, thereby reducing rate of insect reproduction and the occurrence of disease. In addition, CGA reduced the pupation rate, eclosion rate, sex ratio, and the number of eggs laid per female, indicating that dietary CGA can negatively affect larval development and insect reproduction. These results were consistent with the conclusions reached for other lepidopteran insects. For example, egg production by the gypsy moth is inversely proportional to the phenolic acid content of its food (Tod et al., 2000). *Lymantria dispar* larvae could not complete normal growth and development after feeding on an artificial diet containing tannic acid or CGA. The body weights of the larvae were about 67.2%–75.0% lower than that of the control, and the duration of the larval stage was prolonged by 2–4 times. (Wang et al., 2014). This provides new evidence supporting the hypothesis proposed by Caroline and Simon (Caroline and Simon, 2002) that insects can adapt to different foods by adjusting their reproductive capacity during long-term evolution. After being treated with sulfoxaflor at sublethal concentrations, fatty acids, amino acids, and the composition and content of carbohydrates all changed to different degrees, indicating these energy substances mentioned above participate in detoxification metabolism of *M. persicae* to some extent (Zhang et al., 2021). We speculate that the effect of CGA on the growth and development of *M. separata* might be related to some energy substances involved in detoxification metabolism, which needs further research.

### 4.3 The role of P450 genes in CGA detoxification

Plant secondary metabolites can induce changes in detoxification-related protein activities in phytophagous insects, which may improve the adaptability of these insects (Chen et al., 2015; Wang et al., 2022). CYP450 enzymes are the primary detoxifying enzymes in many organisms. Multiple signaling pathways and critical effector molecules are involved in regulating insect P450s. CYP450 genes play a significant role in detoxification in insects, and insecticide resistance largely depends on the metabolism of exogenous toxic substances by CYP450s (Nauen et al., 2021; Lu et al., 2020). Increased P450 activity is a key mechanism inducing insect resistance (Ye et al., 2022). Wang et al. demonstrated that *AmCYP9q1* plays an important role in the metabolic detoxification of imidacloprid by *Apis mellifera* larvae (Wang et al., 2022). The expression levels of *CYP4L13* and *CYP4M14* genes in the midgut and fat bodies of *Spodoptera frugiperda* increased significantly after larvae were fed exogenous insecticides such as nicotine and flavonoids (Wang et al., 2022). Here, we performed assays of P450 enzyme activity in *M. separata* larvae treated with CGA to investigate the role of *MsCYP450* genes. We found that CGA could induce P450 enzyme activity in *M. separata* larvae fed with different concentrations of CGA for different amounts of time (**Figure 3**). Studies have shown that P450 can add various chemical groups, including hydroxyl, carboxyl, and amino groups, to toxic secondary metabolites in the insect digestive tract. They can improve the water solubility and reactivity of toxic secondary metabolites and degrade them into less harmful forms (Chung et al., 2007; Cui et al., 2016). In this study, we found evidence that CYP450 enzymes are essential for the detoxification metabolism of CGA in *M. separata*.

In transcriptome analysis, we found 139 *McCYP450* genes, which is a much higher number than those in the lepidopteran insects *H. armigera* (112) and *S. litura* (67) (Zhang, 2018 and Zhang, 2019). Fewer *MsCYP450* genes were identified in this study than in our previous transcriptome sequencing study (Wang et al., 2018), possibly because of insufficient sequencing depth or redundant sequencing. According to phylogenetic tree analysis, the 139 *MsCYP450* genes were divided into four clans, of which the CYP3 clan was most closely related to drug resistance metabolism (Wan et al., 2013). By comparing the transcriptomes of *M. separata* treated with different concentrations of CGA with that of the control group, 179 commonly up-regulated genes were identified. Among these up-regulated genes, seven were *MsCYP450* genes and five were from the CYP3 clan. Therefore, we speculated that the seven *MsCYP450* genes might be involved in metabolism. To further confirm the function of these genes, we used the RNAi to knock down their expression. Insect RNAi has been widely used to identify or validate insecticide target genes (Young et al., 2015; Dulbecco et al., 2021) and the use of this technology in *M. separeata* has been reported (Wang et al., 2019). Also this method is widely used in identification of insecticide target. In our study, *M. separata* larvae were continuously fed dsRNA for 3 days, and the mRNA level of *MsCYP450* genes were significantly lower after treatment. The surviving larvae were then exposed to the CGA at LC_50_ for 6 days, and treatment with dsMsCYP6B6, dsMsCYP321A7, and dsMsCYP6B7-like caused a significant reduction in survival compared with the CK and the dsGFP treatment groups. These three *MsCYP450* genes all belonged to the CYP3 clan, which indicates they might play an important role in the detoxification of CGA. The same results have also been observed for similar genes in other insects. Bagchi et al. also demonstrated that CGA significantly induces the CYP450 genes of *Amyelois transitella* and increases the tolerance to CGA (Bagchi et al., 2016), and silencing of the cytochrome P450 gene *CYP321A1* was found to affect tannin detoxification in *S. litura* (Zhao et al., 2021). *CYP6B6* was shown to be involved in esfenvalerate detoxification in the polyphagous insect *H. armigera* (Tian et al., 2017), and *CYP6B7* was shown to play an important role in the resistance of *H. armigera* to fenvalerate (Tang et al., 2007). In this study, we confirmed that *MsCYP6B6, MsCYP321A7*, and *MsCYP6B7-like* play an essential role in the detoxification of CGA in *M. separata*.

In this study, we found that CGA had a lethal effect on *M. separata* and that a sublethal concentration harmed larval growth and development. Seven *MsCYP450* genes that may be involved in the detoxification process were identified by performing P450 enzyme assays and transcriptome analysis. By treating larvae with dsMsCYP450 genes, we determined that MsCYP6B6, MsCYP321A7, and MsCYP6B7-like play a vital role in the detoxification of CGA by *M. separata*. The findings of this functional study of the CGA detoxification genes of this major phytophagous insect provides new insight into this biological process and new targets for agricultural pest control. This study also provides a new method for managing P450-mediated resistance in insect pests.

## Data availability statement

The data presented in the study are deposited in the Genebank repository, accession number MsCYP321A7 (OP254196),MsCYP6k1-like(OP254197), MsCYP6B6(OP254198),MsCYP324A1 (OP254199),MsCYP4V2-like(OP254200),MsCYP6B7-like (OP254201),MsCYP6AE88 (OP254202).

## Author contributions

D-jL, L-yL, and L-zZ conducted the experiment, D-jL manuscript writing, YF and S-jG data analysis, RW and J-dWdesigned the experiment and funding support. All authors haveread and agreed to the published version of the manuscript.

## Funding

This work was supported by the Scientific and Technological Innovation Capacity Construction Special Funds of the Beijing Academy of Agriculture and Forestry Sciences, China (KJCX20210437), the key research and development program of Hunan Province (China) (2020NK2034), Talent Programs of Fujian Agriculture and Forestry University (xjq202119), the Sugar Crop Research System, CARS (CARS-17) and Special Technology Innovation Funding of Fujian Agriculture and Forestry University (CXZX2020084A).

## Conflict of interest

The authors declare that the research was conducted in the absence of any commercial or financial relationships that could be construed as a potential conflict of interest.

The reviewer ZG declared a past collaboration RW with the author to the handling editor.

## Publisher’s note

All claims expressed in this article are solely those of the authors and do not necessarily represent those of their affiliated organizations, or those of the publisher, the editors and the reviewers. Any product that may be evaluated in this article, or claim that may be made by its manufacturer, is not guaranteed or endorsed by the publisher.
