# Supplementary material for: The insecticidal effect of the botanical insecticide chlorogenic acid on Mythimna separata (Walker) is related to changes in MsCYP450 gene expression
Source: Front Plant Sci. 2022 Oct 12;13:1015095. doi: 10.3389/fpls.2022.1015095 (PMC9597446; doi:10.3389/fpls.2022.1015095)
Supplement: Supplementary file 1 [file DataSheet_1.pdf]

Table S1 Primers used for qRT-PCR

Gene ID	Gene Name	Primer sequences (5’-3’)
OP254196	*MsCYP321A7*	F:GATGCACGACTTCGCAGATA<br>R:CAAAGCCTGTGCAGACAATAAG
OP254197	*MsCYP6k1-like*	F:CCCAGAGCTGGTGGATATATTTAG<br>R:TCTCATACCCTCCTCTCTGTTC
OP254198	*MsCYP6B6*	F:GGCAGACTTCAGTCCCAATTA<br>R:TCGGAGCAACTCCTAAATTCC
OP254199	*MsCYP324A1*	F:CGCTAACCTGGACTCTGTATG<br>R:GGAGGCATCTAGCTCCTTATTG
OP254200	*MsCYP4V2-like*	F:TTCGAGAACTGTTGCCGTATAG<br>R:TTACTTCGTGCGTAGGAGTTG
OP254201	*MsCYP6B7-like*	F:GGACCGGAACCAACCATATT<br>R:TCTTGCATCTCCACACCTTTAT
OP254202	*MsCYP6AE88*	F:GGAGAACGTAGTCGTCCATAAC<br>R:GTGCACGAAATGCCTTACTTAG
	EF-1α	F:AAGAAATCTGCCCGCGGTAT<br>R:TGCGGTTTAGCGATGGAAGT

Table S2 Primers used in synthesizing dsRNA

Primer name	Primer sequence (5'-3', T7=TAATACGACTCACTATAGGG)
dsMsCYP321A7	F:T7CGGAGTAAACATTTTTGTTC<br>R:T7TTTTCACTGCTGGTGTAGAG
dsMsCYP6k1-like	F:T7TTGTGTAGTCCGTGCAGATT<br>R:T7TCGTCAACAGCCCCGAGATT
dsMsCYP6B6	F:T7AGAAACATGGATGGCCTTAA<br>R:T7GCTTTCAGAAGTTTTGGAAAC
dsMsCYP324A1	F:T7GGCCTTAATTTGTTTACTGTC<br>R:T7TTGAACTTCACAAAGTCCCT
dsMsCYP4V2-like	F:T7AGAAACGAGAAAAGTTGGAA<br>R:T7AGTAATAAACGGTACAGGTG
dsMsCYP6B7-like	F:T7CGTCGAAAACTTTGTTCAAG<br>R:T7GCTCAAAGAAATGGAAAACC
dsMsCYP6AE88	F:T7TGTGTTTACTAAAGATTTCTAC<br>R:T7GTTTCTCTCACATCCATAAC
dsGFP	F:T7AGACAGTGCTTCAGCCGCTAC<br>R:T7GTTCACCTTGATGCCGTTC

Table S3 Bioassay result of the CGA on *M. separata*

	Number	Slope ± SE	LC_{20} (mg/mL) (95% CI)	LC_{50}(mg/mL) (95% CI)	LC_{80}(mg/mL) (95% CI)	χ^2 (df)
2st instar larva	432	1.184±0.392	2.799 (2.112-3.595)	14.343 (11.425-18.084)	73.823 (57.469-97.808)	1.998(13)
3rd instar larva	432	1.469±0.286	7.148 (5.604-8.603)	26.296 (21.852-31.623)	98.352 (80.372-123.391)	2.491(13)
4th instar larva	432	1.728±0.421	13.813 (11.392-16.401)	42.393 (36.224-49.622)	130.107 (109.601-157.789)	0.935(13)

Table S4 List of MsCYP450 with > 200 amino acids in M. separate transcriptome data

Gene id	Amino acid length	E-value	NR description
Cluster-2594.56608	531	3.80E-230	*antennal cytochrome P450 CYP9 [Mamestra brassicae]*
Cluster-2594.39395	503	3.60E-270	*CYP332A1 [Mythimna separata]*
Cluster-2594.49775	556	1.60E-190	*CYP4G200 [Mythimna separata]*
Cluster-2594.54377	529	6.20E-301	*CYP6AE88 [Mythimna separata]*
Cluster-2594.56702	531	4.60E-80	*CYP9A112 [Mythimna separata]*
Cluster-2594.38136	513	2.90E-61	*cytochrome CYP333B3 [Spodoptera littoralis]*
Cluster-2594.29667	516	4.60E-14	*cytochrome CYP340AB1 [Spodoptera littoralis]*
Cluster-2594.34432	509	8.40E-178	*cytochrome CYP340K4 [Spodoptera littoralis]*
Cluster-2594.39111	504	1.30E-244	*cytochrome P450 [Helicoverpa armigera]*
Cluster-2594.9160	510	1.50E-251	*cytochrome P450 302A1 [Mamestra brassicae]*
Cluster-2594.75740	539	2.80E-288	*cytochrome P450 307A1 [Mamestra brassicae]*
Cluster-2594.47610	516	1.70E-204	*cytochrome P450 314A1 [Mamestra brassicae]*
Cluster-2594.13975	422	6.60E-09	*Cytochrome P450 4C1 [Papilio machaon]*
Cluster-2594.32558	493	2.00E-18	*cytochrome P450 4C1-like [Helicoverpa armigera]*
Cluster-2594.32800	500	8.90E-245	*cytochrome P450 4C1-like [Spodoptera litura]*
Cluster-2594.37803	490	5.10E-141	*cytochrome P450 4C1-like [Trichoplusia ni]*
Cluster-2594.15723	490	2.40E-199	*cytochrome P450 4c21-like [Spodoptera litura]*
Cluster-2594.68248	492	6.50E-60	*cytochrome P450 4d8-like [Spodoptera litura]*
Cluster-2594.66432	501	5.70E-135	*cytochrome P450 4V2-like [Spodoptera litura]*
Cluster-2594.59927	641	7.70E-211	*cytochrome P450 6B1-like [Bicyclus anynana]*
Cluster-2594.73787	519	1.10E-229	*cytochrome P450 6B2-like isoform X2 [Spodoptera litura]*
Cluster-2594.70526	519	2.90E-24	*cytochrome P450 6B2-like isoform X3 [Spodoptera litura]*
Cluster-2594.50434	504	4.50E-230	*cytochrome P450 6B7-like [Spodoptera litura]*
Cluster-2594.15806	505	2.50E-160	*cytochrome P450 6k1-like [Spodoptera litura]*
Cluster-2594.71852	501	1.30E-258	*cytochrome P450 6k1-like [Spodoptera litura]*
Cluster-2594.36747	511	4.60E-274	*cytochrome P450 9e2-like [Helicoverpa armigera]*
Cluster-2594.39726	507	1.60E-206	*cytochrome P450 CYP12A2-like [Helicoverpa armigera]*
Cluster-2594.62660	501	9.50E-203	*cytochrome P450 CYP12A2-like [Spodoptera litura]*
Cluster-2594.31139	539	2.30E-272	*cytochrome P450 CYP18A1 [Helicoverpa armigera]*
Cluster-2594.48527	495	8.00E-222	*cytochrome P450 CYP321A7 [Spodoptera frugiperda]*
Cluster-2594.17774	492	1.10E-151	*cytochrome P450 CYP337B1 allele 12 [Helicoverpa armigera]*
Cluster-2594.57930	513	9.00E-256	*cytochrome P450 CYP354A3 [Helicoverpa armigera]*
Cluster-2594.46117	563	1.80E-195	*cytochrome p450 CYP4G74 [Spodoptera exigua]*
Cluster-2594.40488	499	1.10E-234	*cytochrome p450 CYP4M18 [Spodoptera exigua]*
Cluster-2594.58405	492	8.40E-199	*cytochrome P450 CYP4S4 [Mamestra brassicae]*
Cluster-2594.62258	493	1.30E-139	*cytochrome p450 CYP4S8 [Spodoptera exigua]*
Cluster-2594.29402	519	1.40E-40	*cytochrome p450 CYP6AE47 [Spodoptera exigua]*
Cluster-2594.51275	517	1.30E-243	*cytochrome p450 CYP6AN4 [Spodoptera exigua]*
Cluster-2594.14358	504	3.30E-232	*cytochrome P450 CYP6B2v3 [Helicoverpa armigera]*
Cluster-2594.39970	534	4.60E-213	*cytochrome P450 CYP9A3 [Helicoverpa armigera]*
Cluster-2594.24641	493	9.60E-219	*cytochrome P450 CYP9G5 [Helicoverpa armigera]*

Cluster-2594.6923	502	3.60E-219	*cytochrome P450 monooxygenase CYP4M7 [Helicoverpa zea]*
Cluster-2594.55188	532	2.30E-239	*cytochrome P450 monooxygenase CYP9A [Mamestra brassicae]*
Cluster-2594.86988	530	3.70E-122	*cytochrome P450 SE-CYP9A21v3, partial [Spodoptera exigua]*
Cluster-2594.37482	529	5.10E-224	*cytochrome P450-like [Helicoverpa armigera]*

Table S5 Sublethal effects of CGA at LC_{20} on developmental duration of *M. separate* after dsRNA treatment

Treatments	Developmental duration (days)			
	3rd instar larva	4th instar larva	5th instar larva	6th instar larva
CK	3.47±0.02a	2.22±0.03c	1.67±0.05c	6.81±0.11a
dsGFP	3.67±0.06a	2.83±0.04b	2.24±0.06b	6.01±0.08b
dsMsCYP321A-7	3.72±0.09a	3.19±0.10a	2.33±0.02ab	5.49±0.06c
dsMsCYP6k1-like	3.68±0.01a	2.91±0.08ab	2.41±0.04ab	5.56±0.07c
dsMsCYP6B6	3.69±0.07a	3.14±0.06ab	2.25±0.02ab	5.32±0.04c
dsMsCYP324A1	3.61±0.02a	2.81±0.05b	2.36±0.08ab	5.45±0.08c
dsMsCYP4V2-like	3.64±0.04a	2.95±0.07ab	2.48±0.06ab	5.80±0.10bc
dsMsCYP6B7-like	3.71±0.03a	2.85±0.02b	2.53±0.05a	5.43±0.05c
dsMsCYP6AE88	3.70±0.06a	2.92±0.03ab	2.31±0.01ab	5.61±0.07c

Data in the table are mean ± SE, and different small letters following the data in a column show significant difference ($P < 0.05$, One-way ANOVA).

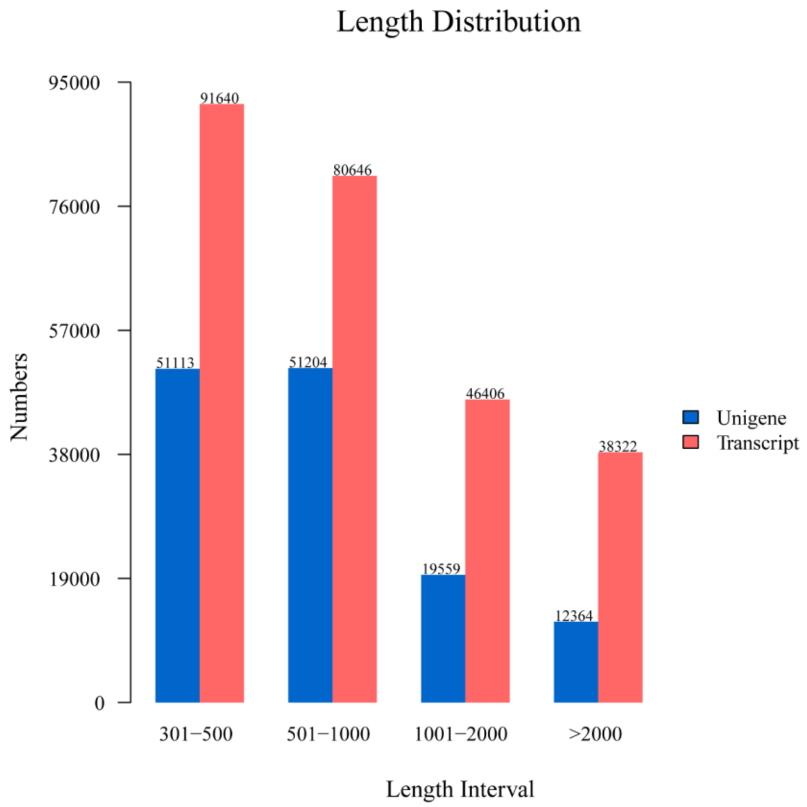

Fig.S1 The length distribution graph of transcripts and unigenes.

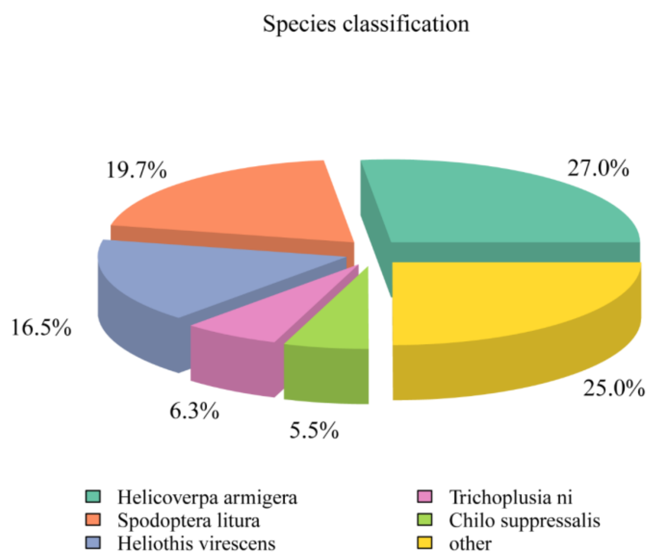

Fig.S2 Species distribution map for unigenes on Nr library alignment

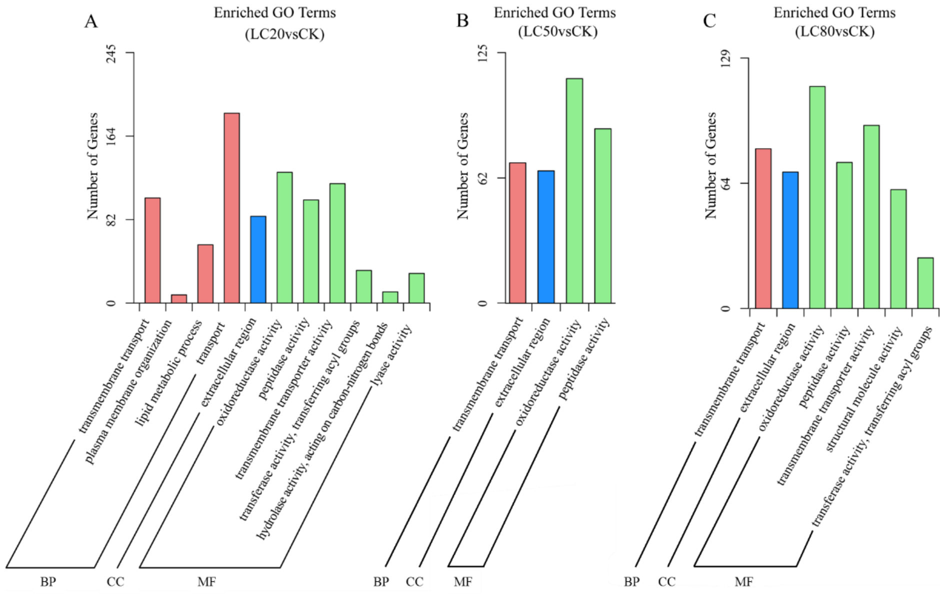

Figure.S3. Functional categorization of DEGs in significantly enriched GO terms.

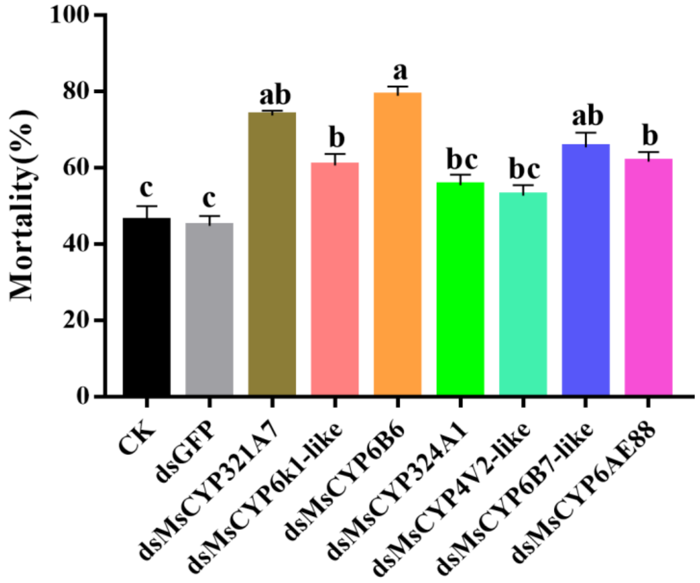

Figure.S4. The larval mortality for 5 days after dsMsCYP450 gene treatment. Different lowercase letters (a, b and c) above the bars indicate significant differences ($p < 0.05$) based on one-way ANOVA followed by Tukey's HSD test for multiple comparisons. Means ± SE from three replicates.
